# Association of lifestyle and sociodemographic factors on multimorbidity: a cross-sectional study in Portugal

**DOI:** 10.1186/s12889-022-14640-5

**Published:** 2022-12-14

**Authors:** Rosália Páscoa, Andreia Teixeira, Hugo Monteiro, Filipe Prazeres, Carlos Martins

**Affiliations:** 1 Medicine Department of Community Medicine, Information and Health Decision Sciences (MEDCIDS), Faculty of Medicine, University of Porto, Al. Prof. Hernâni Monteiro, 4200 - 319 Porto, Portugal; 2 Centre for Health Technology and Services Research (CINTESIS), University of Porto, Al. Prof. Hernâni Monteiro, 4200 - 319 Porto, Portugal; 3 ADiT-LAB, Instituto Politécnico de Viana Do Castelo, Rua Escola Industrial E Comercial Nun’Álvares, 4900-347 Viana Do Castelo, Portugal; 4 Department of Studies and Planning of the Northern Regional Health Administration, Porto, Portugal; 5 Faculty of Health Sciences, University of Beira Interior, Covilhã, Portugal; 6 #H4A Primary Healthcare Research Network, Porto, Portugal

**Keywords:** Lifestyle, Sociodemographic, Multimorbidity

## Abstract

**Background:**

Lifestyle factors are widely recognized as modifiers and major risk factors for non-communicable diseases. Previous studies on the prevalence of multimorbidity in Portugal predict an unfavourable reality. The aim of the present study was to analyse 1) the prevalence of multimorbidity in Portugal and 2) the association of individuals’ lifestyles and sociodemographic factors with multimorbidity.

**Methods:**

A cross-sectional, population-wide study was conducted on a representative sample of the general population of Portuguese adults aged ≥ 20 years. Categorical variables were described by their respective absolute and relative frequencies (n (%)). All variables with a *p*-value < 20% were included in the multiple logistic regression model. The variables were removed one by one in descending order of *p*-value (p) until the model contained only significant variables. The results are presented using the odds ratio and 95% confidence intervals. *P*-values ​​ < 5% were considered significant.

**Results:**

The prevalence of multimorbidity was 48.9% (*n* = 436), and the odds of multimorbidity increased 4% (*p* < 0.001) for each year of increase in age. Participants with reasonable general health status had higher odds of multimorbidity (Odds ratio (OR) = 3.04; *p* < 0.001), and those with poor or very poor general health status had even higher odds (OR = 9.14; *p* < 0.001). Compared to those who never smoked, participants who quit smoking ≥ 1 year presented an increase of 91% (*p* = 0.005) in the odds of multimorbidity. Individuals with no good-quality sleep, non-moderate screen time, or non-moderate stress level had higher odds of multimorbidity (OR = 1.98; OR = 1.88; OR = 2.22, respectively. *p* < 0.001).

**Conclusions:**

This study presented a new approach to multimorbidity in Portugal. Population-based, multidimensional lifestyle interventions are needed. It seems necessary to optimize and adjust measures to prevent non-communicable diseases to improve health in Portugal. In the future, longitudinal studies will be an asset to reinforce and clarify these conclusions.

**Supplementary Information:**

The online version contains supplementary material available at 10.1186/s12889-022-14640-5.

## Background

Lifestyle factors are widely recognized as modifiers and major risk factors for noncommunicable diseases, the leading causes of mortality, disability, impaired quality of life, and global burden on health systems [1–4]. Although individual diseases are the main focus in healthcare delivery, medical education, and research, multimorbidity is becoming the norm in various societies [5]. Multimorbidity is defined as the presence of two or more chronic morbidities and has been receiving broader attention [6, 7]. The European General Practice Research Network also defines multimorbidity as any combination of chronic disease with at least one other disease (acute or chronic), biopsychosocial factor (associated or not), or somatic risk factor [8].

Previous studies on the prevalence of multimorbidity in Portugal predict an unfavourable reality. A cross-sectional epidemiological study with a representative sample of the Portuguese population was carried out between 2013 and 2016 and showed a prevalence of multimorbidity of 38.3% (95% CI: 35.4% to 41.3%) [9]. In that study, the multimorbidity variable was defined as the presence of two or more self-reported chronic diseases based on a list of 20 pathologies. Another analytical, cross-sectional study examined data from Primary Care Centres in Mainland Portugal through a random sample of family doctors from October 2013 to December 2014. The prevalence of multimorbidity (2 or more chronic health problems) was reported as 72.7% [10].

Regarding the association of lifestyle and sociodemographic factors on multimorbidity, the study by Quinaz et al. observed a statistically significant increase in the prevalence of multimorbidity with age. Population groups with higher educational level showed a lower prevalence of multimorbidity, for both genders [9]. In the other Portuguese study, the highest levels of multimorbidity were identified among some vulnerable groups: the elderly, the less educated, and the pensioners/retiree [10]. Neither of the two Portuguese studies evaluated the association of lifestyle with multimorbidity.

Several studies have increasingly explored the association of lifestyle and sociodemographic factors with multimorbidity, given the important relationship that they have and their impact on multimorbidity [7, 11–14]. In Portugal, however, there seem to be no studies on these associations. Thus, the aim of the present study was to analyse 1) the prevalence of multimorbidity in Portugal and 2) the association of individual lifestyles and sociodemographic factors with multimorbidity. This study is relevant to better understanding multimorbidity in Portugal. It presents prevalence data obtained from the general adult population. In addition, it introduces new data on the association between multimorbidity and lifestyle and also complements previous studies on the data related to sociodemographic factors.

## Methods

### Design and setting of the study

A cross-sectional population-wide study was conducted on a representative sample of the general population of Portuguese adults aged 20 years or over. A questionnaire in Portuguese language, whose completion took between 20 and 25 min, was applied in face-to-face interviews at the respondent's home. The exclusion criteria included any cognitive or physical disability that hampered the ability to participate in a face-to-face interview, residence of a collective dwelling, not speaking/understanding Portuguese [15].

### Sample size estimation and sampling techniques

A stratified sampling design was used to obtain a representative sample of the general population of Portuguese adults.

All NUTS II (nomenclature of territorial units for statistical purposes) were used as natural strata, and for each one, a random sample of starting points was selected with a probability proportional to the NUTS population size estimated by the national census [16]. Target quotas were set in consideration of the distribution of the variables gender (male; female), age (groups of every five years except the last defined group), and region of residence (North, Centre, Lisbon, Alentejo, and Algarve). Given the geographical dispersion, interviews were conducted in all district capitals to ensure the proportionality that they represent in the resident population of mainland Portugal.

A sample size of 900 participants was calculated for a 95% confidence level in consideration of the most conservative scenario (*p* = 0.5), an infinite population, and a margin of error of approximately 3. Regarding multiple regression models, the literature recommends 15 participants per independent variable [17]. In this study, we have 13 independent variables, so the sample size of 900 participants meets this recommendation.

Participants were selected using the random route sampling method [18], which implied that each interviewer had an interview number and quotas to reach. The daily visit plan was defined based on a random choice of the street, door number, and floor as the starting point. One individual in each household was selected using the last birthday method (the person selected in each home was the one whose birthday was most recent on the date of the interview). If the quota of the identified individual was fulfilled or the individual did not agree to participate in the study, the previous birthday would be identified. This was done in the same way until the individuals residing in the selected household were exhausted.

If no response was obtained at an address, three new contacts were made on different days and times. If there was no response (or no element could be selected at an address), the address was replaced by another one according to the rules of the random route method. To identify as many people as possible at home, fieldwork was preferably carried out from 5 to 9 pm on weekdays and from 11 am to 9 pm on weekends and holidays.

### Data collection

The data collection was performed from 16 January to 30 April 2019 using a questionnaire in the Portuguese language [15]. The lifestyle factors examined were diet, physical activity, alcohol consumption, tobacco use, illicit drugs, sleep habits, screen time, and stress. Sociodemographic data included age, sex, marital status, highest level of education completed, general health status, and health problems in the last 12 months, among others.

Multimorbidity was measured as the outcome of interest by a simple count of the number of chronic conditions reported by participants from a list of 13 options: osteoarticular/muscular pain, hypertension, anxiety, hypercholesterolemia, overweight, diabetes, heart problems (myocardial infarction, angina pectoris, heart valve disease, heart failure), depression, gastritis or peptic ulcer disease, asthma and/or COPD (chronic bronchitis, chronic obstructive pulmonary disease, or emphysema), stroke, obesity, and cancer. According to their relevance, the list of 13 medical conditions, as determined by the authors, took into account the results of the national health survey (and its methodology regarding the study of general health status) and the information contained in the national health plan [9, 19]. In addition, a practical and feasible approach was favoured, framed in a questionnaire whose main objective was to characterize lifestyles and health behaviours [15]. In our previous study [15], 40.2% (*n* = 358) of participants presented as overweight, and 13.4% (*n* = 119) were obese according to the auto-reported values of weight and height. However, the self-reported prevalence of overweight and obesity was 15.6% (*n* = 140) and 2.1% (*n* = 19), respectively. Given the difference found and the greater objectivity of the prevalence obtained by the body mass index (BMI), the BMI-based prevalence was considered in this study. Furthermore, the prevalence obtained by the BMI ​​was closer to that obtained in a previous national study [20].

The lifestyle variables considered were defined as follows:

- A healthy diet was considered whenever the participant concomitantly ate two to three main meals per day, two to six portions of fruit per day, two or more portions of legumes or salads per day, and had a moderate consumption of alcohol (defined below) [21–23]
- Regular physical activity was defined as walking or doing any physical activity for five or more days of the week and at least 30 to 59 min on average [24]
- Moderate alcohol consumption was defined as a maximum of one drink per day in the case of females and males 65 years or older, while for males under 65 years old, it was considered as a maximum of two drinks per day [25]
- The correct use of tobacco was considered when participants answered “no” to the question, “Do you smoke?” [26]
- The correct use of illicit drugs was considered when participants answered “no” to the question, “Do you use illicit drugs?” [27]
- Good-quality sleep was considered when participants simultaneously selected “few days or never” for the frequency of sleep problems (difficulty falling asleep, sleeping poorly, or oversleeping), as well as for the need to take medication to sleep, along with “most days or always” for having “repairing sleep” in the last two weeks [28, 29]
- Moderate screen time was defined as having fewer than 3 h of screen time on a normal day [30, 31]
- Moderate stress level was considered when participants simultaneously answered “few days or never” about doing less than they wanted in work/daily activities for feeling anxious/nervous, as well as for the frequency of feeling anxious/nervous, and answered “most days or always” for the need for medication to control anxiety and nervousness and feeling calm in the last two weeks [32].

For each lifestyle variable, healthy behaviour was considered as the reference.

### Statistical analysis

Data analysis was performed using SPSS v. 27. Categorical variables were described by their absolute and relative frequencies (n (%)). Simple logistic regressions were performed to determine which variables (sociodemographic and lifestyle variables) were associated with multimorbidity (yes/no). All variables with a *p*-value (p) < 20% were included in the multiple logistic regression model, and then the variables were removed one by one in descending order of *p*-value until the model contained only significant variables [33].

The results of the logistic regression models are presented using the odds ratio (OR) and 95% confidence interval (CI). The suitability of the logistic regression model was verified by the Hosmer–Lemeshow test. *P*-values < 5% were considered significant.

## Results

There were 900 participants who answered the questionnaire, and the sample was representative of the general adult Portuguese population [15]. There were 25 individuals who were excluded for not speaking/understanding Portuguese. As 198 individuals refused to participate, a response rate of about 82% was obtained.

**Fig. 1:**
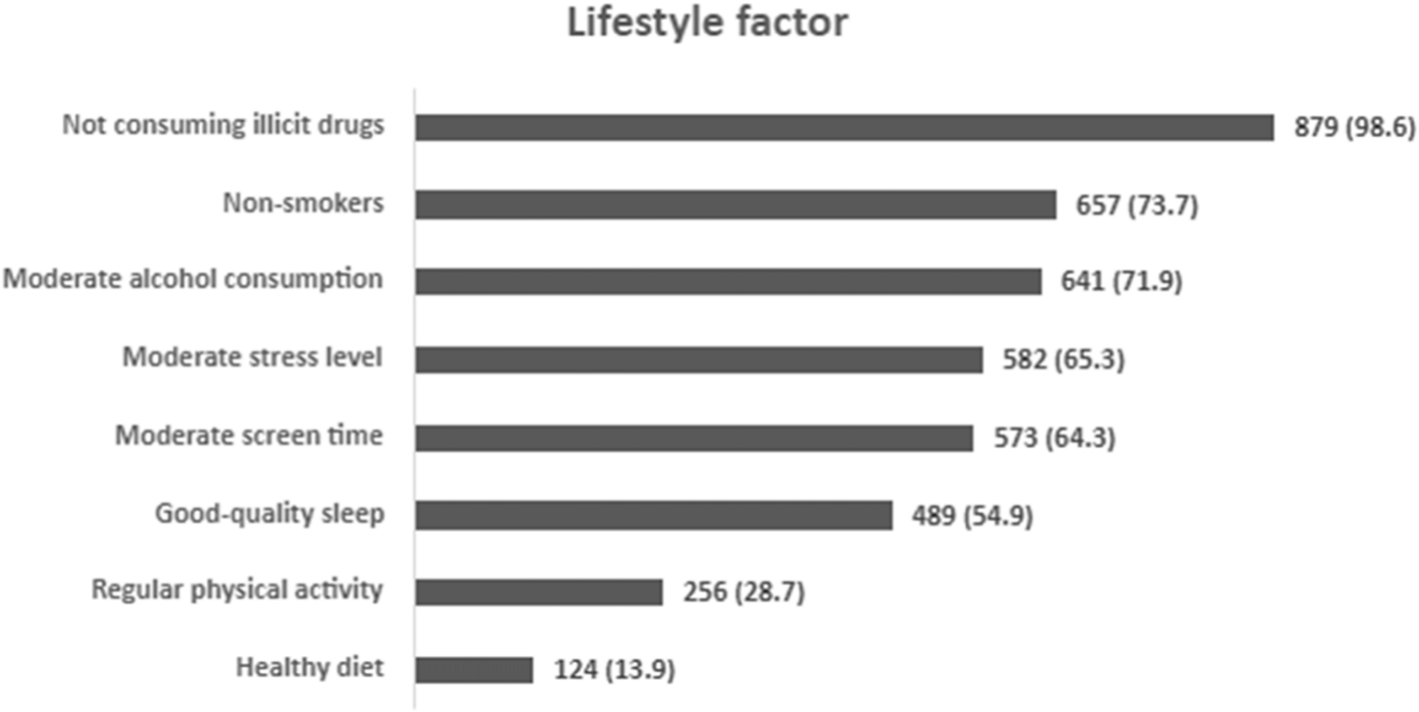
General characterization of the participants’ lifestyle patterns, *n* = 891

Thus, data from 891 participants were considered, after excluding 9 participants whose BMI values ​​were not known, which has a direct impact on the definition of multimorbidity. Table 1 shows a general characterization of the participants' health problems, which indicated a prevalence of multimorbidity of 48.9% (*n* = 436). The numbers and percentages of participants who met the recommendations for each lifestyle behaviour are shown in Fig. 1, and the results of logistic regression analyses for multimorbidity are displayed in Table 2.

**Table 1:** General characterization of the participants’ health problems in the last 12 months

**Health problems, *n* (%)**	
Osteoarticular/muscular pain	349 (38.8)
Hypertension	178 (19.8)
Anxiety	153 (17.0)
Hypercholesterolemia	145 (16.1)
Diabetes	99 (11.0)
Heart problems	72 (8.0)
Depression	58 (6.4)
Gastritis or peptic ulcer disease	49 (5.4)
Asthma and/or COPD	40 (4.4)
Stroke	16 (1.8)
Cancer	12 (1.3)
Overweight, *n* = 891^a^	358 (40.2)
Obesity, *n* = 891^a^	119 (13.4)
None, *n* = 891^a^	206 (23.1)
**Number of health problems**^**b**^**, *n* (%)**	
1	249 (27.9)
2	188 (21.1)
3	108 (12.1)
4	69 (7.7)
≥ 5	71 (8.0)

*COPD* Chronic obstructive pulmonary disease

^a^To calculate the frequency of overweight and obesity, only 891 individuals were considered (out of a total of 900) due to missing values of weight and/or height. Thus, for the calculation of "none” (absence of disease), *n* = 891 was also considered

^b^The most frequent combinations (TOP 5) in each group of various health problems (2, 3, or 4) are shown in Additional file 1

**Table 2:** Multiple logistic regression odds ratios (with confidence intervals) of significant sociodemographic, general health status, and lifestyle behaviours variables, for the outcomes of multimorbidity

	**Unadjusted model**	**Adjusted model**
***N* = 891**	**OR [95% CI]**	**OR [95% CI]**
**General health status**		
Very good or good	Reference	
Reasonable	3.03 [2.11; 4.34]	3.04 [2.13; 4.33]
Poor or very poor	9.09 [3.94; 21.00]	9.14 [3.98; 20.99]
**Moderate stress level**		
Yes	Reference	
No	2.17 [1.48; 3.19]	2.22 [1.52; 3.24]
**Good-quality sleep**		
Yes	Reference	
No	2.03 [1.42; 2.90]	1.98 [1.39; 2.82]
**Tobacco**		
Never smoked	Reference	
Quit > 1 year	2.00 [1.25; 3.19]	1.91 [1.22; 2.99]
Smoke or quit < 1 year	1.21 [0.80; 1.83]	1.15 [0.78; 1.70]
**Moderate screen time**		
Yes	Reference	
No	2.07 [1.44; 2.95]	1.88 [1.33; 2.66]
**Age**		
	1.04 [1.03; 1.05]	1.04 [1.03; 1.05]
**Gender**		
Female	Reference	
Male	0.95 [0.67; 1.36]	
**Highest level of education completed**		
Secondary education or less	Reference	
Higher Education	0.67 [0.41; 1.12]	
**Regular physical activity**		
Yes	Reference	
No	0.80 [0.56; 1.16]	
**Moderate alcohol consumption**		
Yes	Reference	
No	0.78 [0.53; 1.15]	
**Drugs**		
Never used	Reference	
Stop > 1 year	1.04 [0.48; 2.25]	
Use or stop < 1 year	0.31 [0.05; 2.04]	

Marital status and healthy diet did not result in significant variables (*p* > 0.200) in the simple models for multimorbidity (Additional file 2). All the other variables were included in multiple logistic regression models (Table 2). The final multivariate model (Table 2) resulted in an adequate fit of the estimated values and those predicted by the model according to the Hosmer and Lemeshow test of fit (*p* = 0.144). The odds of having multimorbidity increased 4% (*p* < 0.001) for each year of increase in the age of the participant, starting at 20 years of age. Regarding general health status, participants with reasonable general health status had higher odds of experiencing multimorbidity than participants with good or very good general health status (OR = 3.04; *p* < 0.001). Similarly, participants with poor or very poor general health status presented even higher odds of experiencing multimorbidity (OR = 9.14; *p* < 0.001).

Participants who quit smoking for more than 1 year presented an increase of 91% (*p* = 0.005) in the odds of having multimorbidity in comparison to those who never smoked. Participants with no good-quality sleep, non-moderate screen time, or non-moderate stress level had higher odds of multimorbidity than those without each of these behaviours (OR = 1.98; OR = 1.88; OR = 2.22, respectively. *p* < 0.001).

## Discussion

This study presented a new approach of the prevalence of multimorbidity in Portugal, as well as of the association of lifestyle and sociodemographic factors with multimorbidity. To our knowledge, the only two previous Portuguese studies on the prevalence of multimorbidity presented prevalences of 38.3% (95% CI: 35.4% to 41.3%) [9] and 72.7% [10]. Despite the apparent numerical difference, the prevalence of multimorbidity in the present study may agree with the values of both studies.

Although the prevalence found was higher than 38.3%, that value was obtained from the First National Health Examination Survey, which considered a target population aged between 25 and 74 years [9]. It is possible that the highest value found in the present study is due in large part to the fact that it considered a population aged 20 years or more. Therefore, individuals over 75 years of age were included, whereas they were excluded in the study that reported prevalence of 38.3%. The study that found a prevalence of 72.7% despite using the population of people over 18 years old (an age range more like that in this study) used recruitment through general practitioners. The data obtained refer to the adult population of primary-care patients in Portugal, and the multimorbidity was based on the general knowledge of the physician about the patient's history, the patient's self-report, and the medical records. This study could possibly represent the most adequate assessment of multimorbidity in the adult Portuguese population, which makes it very pertinent in quantifying the dimension of the problem.

One population-based study looked at community-dwelling adults aged 50 years and over, which involved 15 European countries. In that study, Portugal had one of the highest prevalences of multimorbidity with prevalence values ​​similar to those found in the present study [12]. Therefore, the present study represents the most adequate assessment of multimorbidity in the adult population in Portugal. Furthermore, it responds to issue raised in a previous study about the need and relevance of understanding the current pattern of multimorbidity in Portugal through a population-based study with a wide age range [9].

The statistically significant increase in the prevalence of multimorbidity with age is in agreement with the results of other Portuguese studies [9, 12]. These data do not seem surprising since the increase in average life expectancy and addition of healthy years of life have been particularly associated with the presence of several non-communicable diseases in the same individual and, therefore, with multimorbidity [34]. Furthermore, as expected, multimorbidity was more likely in those with worse the general health status reported by the individual. Multimorbidity appears to have a major negative impact on an affected person's life. All of this may be related to the shift from the situation of premature mortality to the burden of the years lived with a disability [34].

Other studies have shown that being a current or past smoker was also associated with a higher likelihood of multimorbidity in men, but not in women [11]. In this study, an increase in the odds of multimorbidity was found in those “having quit smoking for more than 1 year”, but not in those who were “smoking or had quit for less than 1 year”. At first glance, these results were not as expected: there were higher odds of multimorbidity for those who still smoked or who quit for less than 1 year. However, one interpretation of these findings may be that most of the participants only quit smoking after having been diagnosed with certain morbidities. Furthermore, recent Portuguese data showed a relevant asymmetry in the prevalence of tobacco consumption in the resident Portuguese population, with values ​​of 42% in the age group of 25–34 years and 10.3% in the age group of 65–74 years [35].

Regarding the association of individual lifestyles and sociodemographic factors with multimorbidity, the results are in line with several studies that have shown that poor sleep quality appears to be associated with cardiometabolic problems (e.g. hypertension, diabetes, obesity, cardiovascular disease), mental disorders (e.g. depression), and mortality [29, 36–38]. Excessive screen time has also been associated with an increased risk of multimorbidity [30]. Furthermore since this behavioural factor can even be combined with other unhealthy factors (e.g. inadequate diet and low levels of physical activity), the association of moderate screen time with lower odds of multimorbidity seems to be expected. The results regarding stress also agree with previous studies and support the association between lower levels of perceived stress and fewer numbers of chronic conditions [13, 39].

The results draw attention to the challenge of multimorbidity and highlight some factors that could have the greatest possible impact: quitting smoking, good-quality sleep, moderate screen time, and adequate stress management.

One of the limitations of this study is that its cross-sectional design did not allow us to establish a causal relationship between the studied variables and multimorbidity. Furthermore, although self-report is the most feasible method for population-based studies, it has the potential to underestimate prevalence and may result in some misclassification [14]. Since the interviews were carried out in all district capitals, the authors admit that some areas (within rural or urban areas), further away from the district capital, may not be as well represented. Regarding the measurement of multimorbidity, this study considered a list of 13 options, which may have influenced the prevalence estimates. Longer lists appear to result in higher estimates of multimorbidity prevalence [40, 41]. The simple dichotomous categorization of some lifestyle factors may have underestimated the true effect of some risk factors due to the loss of information on possible gradual associations between some of the lifestyle factors and the occurrence of chronic conditions, which is a situation that has already been pointed out in other studies [11].

## Conclusions

It was found that about half of the population had multimorbidity criteria. Increasing age, reasonable or worse general health status, and having quit smoke for more than 1 year were the factors that increased the odds of multimorbidity. On the other hand, good-quality sleep, moderate screen time, and moderate levels of stress were less associated with multimorbidity. The results of this study suggest that the burden of multimorbidity in Portugal is excessive. It seems necessary to optimize and adjust measures to prevent non-communicable diseases to improve the health of the Portuguese population. In the future, longitudinal studies will help to reinforce and clarify these conclusions.

## Supplementary Information

**Additional file 1. **The most frequent combinations (TOP 5) in each group of various health problems (2, 3, or 4).

**Additional file 2: Table S1.** Simple logistic regression odds ratios (with confidence intervals and *p*-values) of significant socio-demographic, general health status, and lifestyle behaviours variables for the outcome of multimorbidity.

## Data Availability

The datasets analysed in the current study are available from the corresponding author on reasonable request.

## Abbreviations

BMI: Body mass index
COPD: Chronic obstructive pulmonary disease
