# Supplementary material for: Association of lifestyle and sociodemographic factors on multimorbidity: a cross-sectional study in Portugal
Source: BMC Public Health. 2022 Dec 14;22:2341. doi: 10.1186/s12889-022-14640-5 (PMC9749348; doi:10.1186/s12889-022-14640-5)
Supplement: Supplementary file 1 — Additional file 1. The most frequent combinations (TOP 5) in each group of various health problems (2, 3, or 4). [file 12889_2022_14640_MOESM1_ESM.docx]

Additional file 1.

The most frequent combinations (TOP 5) in each group of various health problems (2, 3, or 4).

| 2 Comorbidities (n=188), *n* (%) | |
| --- | --- |
| Osteoarticular/muscular pain & Overweight or Obesity, 61 (32.4) |  |
| Anxiety & Overweight or Obesity, 13 (6.9) |  |
| Hypertension & Overweight or Obesity, 11 (5.8) |  |
| Hypercholesterolemia & Overweight or Obesity, 11 (5.8) |  |
| Osteoarticular/muscular pain & Anxiety, 11 (5.8) |  |
| Diabetes & Osteoarticular/muscular pain, 9 (4.8) |  |
| Hypercholesterolemia & Osteoarticular/muscular pain, 8 (4.2) |  |

| 3 Comorbidities (n=108), *n* (%) | |
| --- | --- |
| Hypercholesterolemia & Osteoarticular/muscular pain & Overweight or Obesity, 13 (12.0)  Hypertension & Osteoarticular/muscular pain & Overweight or Obesity, 11 (10.2)  Osteoarticular/muscular pain & Anxiety & Overweight or Obesity, 10 (9.3)  Diabetes & Osteoarticular/muscular pain & Overweight or Obesity, 5 (4.6)  Osteoarticular/muscular pain & Gastritis/peptic ulcer & Overweight or Obesity, 5 (4.6)  Hypertension & Hypercholesterolemia & Overweight or Obesity, 4 (3.7)  Anxiety & Depression & Overweight or Obesity, 4 (3.7)  Hypertension & Osteoarticular/muscular pain & Anxiety, 4 (3.7) |  |

| 4 Comorbidities (n=69), *n* (%) | |
| --- | --- |
| Hypertension & Hypercholesterolemia & Osteoarticular/muscular pain & Overweight or Obesity, 10 (14.5)  Hypercholesterolemia & Diabetes & Osteoarticular/muscular pain & Overweight or Obesity, 5 (7.2)  Hypertension & Diabetes & Osteoarticular/muscular pain & Overweight or Obesity, 5 (7.2)  Hypertension & Hypercholesterolemia & Diabetes & Overweight or Obesity, 4 (5.8)  Hypertension & Osteoarticular/muscular pain & Anxiety & Overweight or Obesity, 4 (5.8)  Hypertension & Osteoarticular/muscular pain & Gastritis/peptic ulcer & Overweight or Obesity, 3 (4.3)  Hypertension & Hypercholesterolemia & Heart problems & Overweight or Obesity, 2 (2.9)  Hypertension & Osteoarticular/muscular pain & Asthma and/or COPD & Overweight or Obesity, 2 (2.9)  Hypertension & Osteoarticular/muscular pain & Anxiety & Depression, 2 (2.9)  Hypertension & Heart problems & Osteoarticular/muscular pain & Gastritis/peptic ulcer, 2 (2.9)  Hypertension & Gastritis/peptic ulcer & Anxiety & Overweight or Obesity, 2 (2.9)  Hypertension & Hypercholesterolemia & Anxiety & Overweight or Obesity, 2 (2.9)  Hypercholesterolemia & Heart problems & Osteoarticular/muscular pain & Overweight or Obesity, 2 (2.9)  Heart problems & Osteoarticular/muscular pain & Anxiety & Overweight or Obesity, 2 (2.9)  Osteoarticular/muscular pain & Asthma and/or COPD & Depression & Overweight or Obesity, 2 (2.9)  Osteoarticular/muscular pain & Anxiety & Depression & Overweight or Obesity, 2 (2.9) |  |
