# Supplementary material for: Association of lifestyle and sociodemographic factors on multimorbidity: a cross-sectional study in Portugal
Source: BMC Public Health. 2022 Dec 14;22:2341. doi: 10.1186/s12889-022-14640-5 (PMC9749348; doi:10.1186/s12889-022-14640-5)
Supplement: Supplementary file 2 — Additional file 2: Table S1. Simple logistic regression odds ratios (with confidence intervals and p-values) of significant socio-demographic, general health status, and lifestyle behaviours variables for the outcome of multimorbidity. [file 12889_2022_14640_MOESM2_ESM.docx]

Additional file 2.

Table S1. Simple logistic regression odds ratios (with confidence intervals and p-values) of significant socio-demographic, general health status, and lifestyle behaviours variables for the outcome of multimorbidity.

| N = 891 | **OR [95% CI]** | **p-value** |
| --- | --- | --- |
| **Gender** | | |
| Female, n = 478 | Reference | |
| Male, n = 413 | 0.71 [0.54; 0.92] | 0.010* |
| **Age** | 1.05 [1.04; 1.06] | <0.001* |
| **Highest level of education completed** | | |
| Secondary education or less, n = 776 | Reference | |
| Higher education, n = 115 | 0.47 [0.31; 0.71] | <0.001* |
| **Marital status** | | |
| Married, n = 501 | Reference | |
| Others, n = 390 | 1.02 [0.78; 1.33] | 0.876 |
| **General health status** | | |
| Very good or good, n = 491 | Reference | |
| Reasonable, n = 322 | 5.76 [4.23; 7.84] | <0.001* |
| Poor or very poor, n = 78 | 21.72 [10.19; 46.31] | <0.001* |
| **Healthy diet** | | |
| Yes, n = 124 | Reference | |
| No, n = 767 | 0.95 [0.65; 1.39] | 0.798 |
| **Regular physical activity** | | |
| Yes, n = 256 | Reference | |
| No, n = 635 | 1.25 [0.94; 1.68] | 0.129 |
| **Moderate alcohol consumption** | | |
| Yes, n = 641 | Reference | |
| No, n = 250 | 0.65 [0.48; 0.87] | 0.004* |
| **Tobacco** | | |
| Never smoked, n = 507 | Reference | |
| Quit > 1year, n = 150 | 1.96 [1.34; 2.86] | <0.001* |
| Smoke or quit < 1 year, n = 234 | 0.72 [0.52; 0.98] | 0.039* |
| **Drugs** | | |
| Never used, n = 837 | Reference | |
| Stop > 1 year, n = 42 | 0.84 [0.45; 1.57] | 0.583 |
| Use or stop < 1 year, n = 12 | 0.20 [0.04; 0.93] | 0.041* |
| **Good-quality sleep** | | |
| Yes, n = 489 | Reference | |
| No, n = 402 | 3.67 [2.78; 4.85] | <0.001* |
| **Moderate screen time** | | |
| Yes, n = 573 | Reference | |
| No, n = 318 | 1.75 [1.33; 2.31] | <0.001* |
| **Moderate stress level** | | |
| Yes, n = 582 | Reference | |
| No, n = 309 | 3.27 [2.44; 4.37] | <0.001* |

*: significant <5%.
